# Impact of rice cultivar and organ on elemental composition of phytoliths and the release of bio-available silicon

**DOI:** 10.3389/fpls.2014.00529

**Published:** 2014-10-10

**Authors:** Zimin Li, Zhaoliang Song, Jean-Thomas Cornelis

**Affiliations:** ^1^School of Environment and Resources, Zhejiang Agricultural and Forestry UniversityLin'an, China; ^2^State Key Laboratory of Environmental Geochemistry, Institute of Geochemistry, Chinese Academy of SciencesGuiyang, China; ^3^Soil Science and Environment Geochemistry, Earth and Life Institute, Université Catholique de LouvainLouvain-la-Neuve, Belgium

**Keywords:** phytolith, rice, silicon solubility, PhytOC, soil-plant systems

## Abstract

The continental bio-cycling of silicon (Si) plays a key role in global Si cycle and as such partly controls global carbon (C) budget through nutrition of marine and terrestrial biota, accumulation of phytolith-occluded organic carbon (PhytOC) and weathering of silicate minerals. Despite the key role of elemental composition of phytoliths on their solubility in soils, the impact of plant cultivar and organ on the elemental composition of phytoliths in Si high-accumulator plants, such as rice (*Oryza sativa*) is not yet fully understood. Here we show that rice cultivar significantly impacts the elemental composition of phytoliths (Si, Al, Fe, and C) in different organs of the shoot system (grains, sheath, leaf and stem). The amount of occluded OC within phytoliths is affected by contents of Si, Al, and Fe in plants, while independent of the element composition of phytoliths. Our data document, for different cultivars, higher bio-available Si release from phytoliths of leaves and sheaths, which are characterized by higher enrichment with Al and Fe (i.e., lower Si/Al and Si/Fe ratios), compared to grains and stems. We indicate that phytolith solubility in soils may be controlled by rice cultivar and type of organs. Our results highlight that the role of the morphology, the hydration rate and the chemical composition in the solubility of phytoliths and the kinetic release of Si in soil solution needs to be studied further. This is central to a better understanding of the impact of soil amendment with different plant organs and cultivars on soil OC stock and on the delivery of dissolved Si as we show that sheath and leaf rice organs are both characterized by higher content of OC occluded in phytolith and higher phytolith solubility compared to grains and stems. Our study shows the importance of studying the impact of the agro-management on the evolution of sinks and sources of Si and C in soils used for Si-high accumulator plants.

## Introduction

Silicon (Si), the second most abundant element in the continental surface (Wedepohl, 1995), is present in soils mostly as silicates, adsorbed Si onto oxides, biogenic precipitates called phytoliths and microorganisms remains. Dissolved Si (H_4_SiO_4_), can be readily taken up by plants and plays an important role as an alleviator of both biotic and abiotic stress (Epstein, 1994; Marschner, 1995; Mecfel et al., 2007). More importantly, Si is usually coupled with carbon (C) in different terrestrial biogeochemical processes (silicate weathering, soil formation, biota nutrition) that occur at different time-scales, which plays a crucial role in the regulation of atmospheric CO_2_ (Oldenburg et al., 2008; Street-Perrott and Barker, 2008; Li et al., 2011; Song et al., 2012).

Si taken up by roots is mainly deposited in the transpiration sites of plants (e.g., cell walls, cell lumina, and intercellular spaces typically near evaporating surfaces) where polymerization of hydrated amorphous silica occurs to form phytoliths (Piperno, 1988; Ma, 2003). The chemical compositions of phytoliths consists mainly of SiO_2_ (66–91%), organic carbon OC (1–6%), H_2_O (0–11%), Al (0.01–4.55%), and Fe (0–2.1%) (Jones and Milne, 1963; Wilding, 1967; Wilding et al., 1967; Wang and Lü, 1993; Wang, 1998; Blecker et al., 2006). The chemical composition of phytoliths is highly dependent on plant species (Kameník et al., 2013). Despite their higher solubility compared to silicate minerals (Fraysse et al., 2009, 2010), phytoliths can be highly resistant to dissolution in specific physico-chemical conditions and can remain for thousands of years in soils (Wilding, 1967; Parr and Sullivan, 2005). Therefore, plant phytoliths play a major role in palaeobotanical, palaeoecological and archeological reconstructions (Clarke, 2003; Piperno, 2006; Cao et al., 2007; Raven and Giordano, 2009). Recent studies report that OC (0.2–5.8%) can be occluded in phytoliths (PhytOC) during plant growth (Parr and Sullivan, 2005; Parr et al., 2010; Li et al., 2013). Si polymerized in plant phytoliths may be more soluble compared to other crystalline mineral phases (Fraysse et al., 2009, 2010), while PhytOC is relatively more stable than other OC fractions in soils (Parr and Sullivan, 2005). For example, Parr and Sullivan (2005) report that the age of phytoliths in volcanic soils and peatland sediments ranges from 0 to 8000 year BP. Moreover, it has been reported that phytoliths in some sediments have a radiocarbon date of 13,300 ± 450 year BP (Wilding, 1967). PhytOC, a crucial component of terrestrial C sink, can represent up to 82% of total C in some soils and sediments after 2000 years of litter fall decomposition, contributing 15–37% in long term terrestrial C sequestration (Alexandre et al., 1997; Parr and Sullivan, 2005).

Cultivated rice (*Oryza sativa*) is the main food source for more than 50% of the global population (Salekdeh et al., 2002) and is currently cultivated on around 1.55 × 10^8^ ha (i.e., 1.04% of the global terrestrial surface) (IRRI, 2011)^1^. Rice, a typical Si high-accumulator, can accumulate more than 10% of SiO_2_ in dry matter (Alvarez and Datnoff, 2001) and application of Si may improve rice growth both under greenhouse and field conditions (Epstein, 1994, 1999; Liang et al., 2007). Recent studies showed that rice plays a significant role in the coupled terrestrial biogeochemical cycles of C and Si through the production of PhytOC (Parr et al., 2010; Song et al., 2012; Li et al., 2013). However, the impact of rice cultivar and of different rice organs on the phytolith elemental composition and solubility has never been reported (Savant et al., 1997), while solubility studies are important to make correct estimates of PhytOC preservation in soils and Si release in soil solution.

In this study, we investigated elemental compositions of organs and phytoliths, and the release of bio-available Si from rice phytoliths of different cultivars and organs (leaves, sheaths, stems and grains) cultivated in identical pedo-climatic conditions (Hortic Anthrosol in subtropical humid climate), to better understand the role of rice cultivars and organs on elemental composition (Si, Al, Fe, C) of phytolith and the release of bio-available Si (solubility in CaCl_2_ solution at 0.01 M). Our results offer a research perspective to further study how to improve the phytolith C sequestration and Si fertility in soil-plant ecosystem.

## Materials and methods

### Sampling sites

Organ samples (leaves, sheaths, stems and grains) of five rice cultivars (three replicates) were collected during the harvest season in October 2010, from the regional trials of new varieties of crops grown at the experimental site of Zhejiang Soil and Fertilizer Station (30°56′06.3″N and 120°51′52.9″E) in Jiaxing City, Zhejiang Province, southeast China. The experimental site is located in Hangjiahu Plain which experiences a typical subtropical humid monsoon climate, with an average annual precipitation of 1200 mm. The mean annual temperature is 16°C and the number of frost free days is 230. Soil is classified as the Gleysols (FAO, 1974)^2^.

### Plant sample preparation and analysis

The five rice cultivars planted in the experiment were Jiahua-11, Xianghu-301, Zhejing-37, Ning-81, and Xiushui-09 which have been grown there for 8 years under identical pedo-climatic conditions to eliminate factors that might influence Si uptake and deposition (Table 1). We collected 8–10 rice cultivars from each site and their associated surface soil (0–5 cm). Then the stem, leaf, sheath and grains of each rice cultivar plant were separated. The sample material was thoroughly washed with ultrapure water after an ultrasonic bath for 15 min, dried at 75°C for 48 h. The dried and mixed samples were separated into two subsamples. One subsample was crushed to determine total elemental composition. Total Si, Al and Fe contents in plant samples and in a plant standard GBW 07602 (GSV-1) were determined by ICP-AES after combustion at 950°C followed by borate fusion (Lu, 2000).

**Table 1:** **Physicochemical soil parameters in the soil surface (0–5 cm) for the different rice species cultivated in the experimental site**.

**Rice cultivars**	**pH**	**SOC**	**SiO_2_**	**Fe_2_O_3_**	**Al_2_O_3_**	**Phytoliths**
				**g/kg**		
Jiahua-11	5.8 ± 0.2a	23.4 ± 1.0b	661.1 ± 41.1a	279 ± 18.8a	142 ± 3.7a	19.2 ± 3.2a
Xianghu-301	5.8 ± 0.1a	15.2 ± 0.7c	681.4 ± 49.2a	286.5 ± 12.1a	146.2 ± 3.5a	11.6 ± 2.1b
Zhejing-37	6.0 ± 0.2a	24.8 ± 0.5b	660.6 ± 23.4a	271.9 ± 1.8a	147.1 ± 3.5a	15.3 ± 1.3ab
Ning-81	6.0 ± 0.0a	22.6 ± 0.4b	661.7 ± 37.4a	260.5 ± 11.3a	146.8 ± 2.8a	16.9 ± 2.1ab
Xiushui-09	5.8 ± 0.1a	29.3 ± 0.7a	670.2 ± 46.9a	281.2 ± 10.1a	146.10.5a	15.1 ± 1.0ab
**Mean**	**5.9a**	**22.9b**	**667.0a**	**275.8a**	**145.7a**	**15.6ab**

*Means with various letters are significantly different at the p < 0.05 level of confidence according to Duncan's Multiple Range Test*.

The other subsample was cut into small pieces around 5 mm to extract phytoliths. Using a microwave digestion method (Parr and Sullivan, 2011) and Walkley-Black type digest (Walkley and Black, 1934), we extracted the phytoliths from all rice organs and thoroughly removed extraneous organic materials in the samples (Li et al., 2013). The phytoliths extracted were oven-dried at 75°C to a constant weight. The organic C released from phytoliths after HF treatment was dried at 45°C and the C content was determined by classical potassium dichromate method (Lu, 2000; Li et al., 2013). The organic C data were monitored with a standard reference soil of 30 mg (GBW07405). The precision is better than 7%.

Phytoliths samples were dissolved in an alkaline solution (NaOH, 1 M) (Saccone et al., 2006), in which the contents of Si, Al, and Fe were analyzed by ICP-AES.

### Bio-available Si analysis

The pool of “bio-available Si” was determined by a CaCl_2_ extraction (0.01 M) (Haysom and Chapman, 1975; Buck et al., 2010; Narayanaswamy and Prakash, 2010) on dried phytolith samples. 30 mg of phytoliths was shaken in 50 ml of CaCl_2_ solution at 20°C for 5 h, 1–9 days. At the end of each extraction, the concentration of Si was analyzed by ICP-AES. The amount of Si released in the extract over time represents the evolution of the immediately available Si fraction of the readily soluble Si pool (Berthelsen et al., 2001; Sauer et al., 2006; Cornelis et al., 2011). This kinetic extraction (expressed as mg bioavailable Si/g SiO_2_ in phytoliths) has been designed to study the potential of phytoliths present in rice cultivars to replenish the bio-available Si pool (Houben et al., 2014).

### Soil samples preparation and analysis

Soil pH was measured in a 1:5 soil: water suspension. Soil organic carbon (SOC) was determined by wet digestion method with 133 mol L^−1^ K_2_Cr_2_O_7_ and concentrated H_2_SO_4_ at 170–180°C. Total SiO_2_, Fe_2_O_3_, and Al_2_O_3_ in soil were measured by ICP-AES after calcinations at 950°C followed by borate fusion (Lu, 2000). Soil phytolith extraction methods were slightly modified from methods of Piperno (1988) and Lu et al. (2006) as follows: Na_4_P_2_O_7_ (20%) deflocculating, treatment with H_2_O_2_ (30%), and cold HCl (15%), ZnBr_2_ heavy liquid (2.38 g-cm^3^) separation. At the end of the soil extraction, the supernatant was separated from the solid residue by centrifugation (3000 g; 5 min) for analyzing phytolith content. All phytoliths extracted were oven-dried at 75°C and weighed to calculate their contents.

### Statistical analysis

All data are presented as the average of three analytical replicates (soil and plant samples). A One-Way analysis of variation (ANOVA) was carried out on the data obtained from the present study, and means were compared using Duncan's Multiple Range Test (*p* < 0.05). The statistical analyses were carried out with SAS software (SAS Institute, 1989).

## Results

### Physico-chemical soil characteristics

As shown in Table 1, the pH value (5.9) was similar in the soil surface of the five different rice species. The total concentration of Fe_2_O_3_ and Al_2_O_3_ were also identical in the soil surface of the five experimental plots, with mean value at 276 and 146 g/kg, respectively. The total concentration of SiO_2_ was also identical in the soil surface of the five experimental plots, with mean value at 667 g/kg. The concentration of SOC and phytoliths varied from 15 to 29 g/kg (mean at 23 g/kg) and from 12 to 19 g/kg (mean at 16 g/kg), respectively. This variation can be explained by the fact that the cultivation of different rice cultivars may cause differences in terms of straw production and residue input in soils, which can influence significantly the content of phytoliths and organic matter in soil.

### Phytolith content and chemical composition of rice organs of different cultivars

The average content of phytoliths in the five rice cultivars varied significantly from 2.2 to 12.0% between rice organs (Table 2). Generally, the phytolith content decreased from 12.0% in sheaths >7.0% in leaves >3.7% in stems >2.2% in grains. The average of Al content for different organs in the five rice cultivars varied from 0.003 to 0.009% (sheaths > leaves = stems = grains). The Fe content in rice organs varied between 0.012 and 0.034% and showed the following trend: sheaths > stems = leaves = grains. The content of OC occluded in phytoliths (PhytOC) of rice organs varied significantly between 0.06 and 0.23% and showed the following trend: sheaths > leaves > stems > grains.

**Table 2:** **Elemental compositions (%) and phytolith content in organs (grains, sheath, stem and leaf) of the different rice cultivars**.

**Rice cultivars**	**Rice organs**	**Phytolith**	**Si**	**Al**	**Fe**	**PhytOC^a^**
		**%**				
Xiushui-09	Grains	2.06 ± 0.03C	1.21 ± 0.15A	0.004 ± 0.001A	0.013 ± 0.002A	**0.04** ± **0.00AB**
Ning-81		2.66 ± 0.39A	0.98 ± 0.12A	0.003 ± 0.000AB	0.012 ± 0.000A	**0.08** ± **0.02A**
Xianghu-301		2.72 ± 0.58A	0.99 ± 0.02A	0.003 ± 0.000AB	0.012 ± 0.001A	**0.06** ± **0.02AB**
Zhejing-37		1.55 ± 0.58D	0.64 ± 0.06B	0.002 ± 0.001AB	0.013 ± 0.001A	**0.04** ± **0.02B**
Jiahua-11		2.16 ± 0.21B	1.26 ± 0.06A	0.001 ± 0.000B	0.009 ± 0.002A	**0.06** ± **0.01AB**
**Mean**		**2.23d**	**1.00d**	**0.003b**	**0.012b**	**0.06d**
Xiushui-09	Sheath	12.48 ± 0.57B	4.95 ± 0.09AB	0.012 ± 0.001A	0.030 ± 0.005B	**0.25** ± **0.02A**
Ning-81		10.89 ± 0.38D	4.37 ± 0.48B	0.011 ± 0.001A	0.047 ± 0.005A	**0.21** ± **0.01BC**
Xianghu-301		11.86 ± 0.58C	4.38 ± 0.21B	0.009 ± 0.001AB	0.033 ± 0.004B	**0.24** ± **0.02AB**
Zhejing-37		10.31 ± 0.24D	3.89 ± 0.38B	0.006 ± 0.001B	0.027 ± 0.002B	**0.23** ± **0.01ABC**
Jiahua-11		14.40 ± 0.70A	6.41 ± 0.79A	0.006 ± 0.002B	0.031 ± 0.001B	**0.20** ± **0.01C**
**Mean**		**11.99a**	**4.80a**	**0.009a**	**0.034a**	**0.23a**
Xiushui-09	Leaf	7.46 ± 0.01B	3.51 ± 0.32B	0.007 ± 0.002A	0.020 ± 0.004A	**0.14** ± **0.00B**
Ning-81		6.46 ± 0.92C	3.76 ± 0.25B	0.006 ± 0.001A	0.019 ± 0.003A	**0.16** ± **0.03B**
Xianghu-301		7.79 ± 0.52A	3.72 ± 0.23B	0.005 ± 0.001A	0.022 ± 0.001A	**0.17** ± **0.02AB**
Zhejing-37		7.93 ± 0.52A	3.37 ± 0.16B	0.004 ± 0.001A	0.022 ± 0.001A	**0.20** ± **0.02A**
Jiahua-11		7.46 ± 0.01AB	4.99 ± 0.35A	0.003 ± 0.001A	0.014 ± 0.001A	**0.17** ± **0.00AB**
**Mean**		**7.04b**	**3.87b**	**0.005b**	**0.019b**	**0.17b**
Xiushui-09	Stem	3.09 ± 0.34C	2.14 ± 0.07A	0.003 ± 0.000AB	0.029 ± 0.004A	**0.07** ± **0.01C**
Ning-81		3.74 ± 1.16B	1.26 ± 0.07A	0.005 ± 0.000A	0.012 ± 0.002B	**0.07** ± **0.03C**
Xianghu-301		3.91 ± 0.24AB	1.46 ± 0.17B	0.004 ± 0.000AB	0.029 ± 0.004A	**0.13** ± **0.01A**
Zhejing-37		4.02 ± 0.23A	2.14 ± 0.23B	0.004 ± 0.001AB	0.014 ± 0.000B	**0.09** ± **0.01BC**
Jiahua-11		4.00 ± 0.50A	2.08 ± 0.19A	0.002 ± 0.001B	0.014 ± 0.001B	**0.11** ± **0.02AB**
**Mean**		**3.75c**	**1.82c**	**0.004b**	**0.020b**	**0.09c**

*Means with various letters are significantly different at the p < 0.05 level of confidence according to Duncan's Multiple Range Test*.

*Different lowercase and uppercase letters indicate significant differences among the stands in rice organs and rice cultivars*.

a *PhytOC, OC occluded in phytoliths per 100 g of dry matter for each organs*.

### Chemical composition of phytoliths extracted from different organs

**Figure 1:**
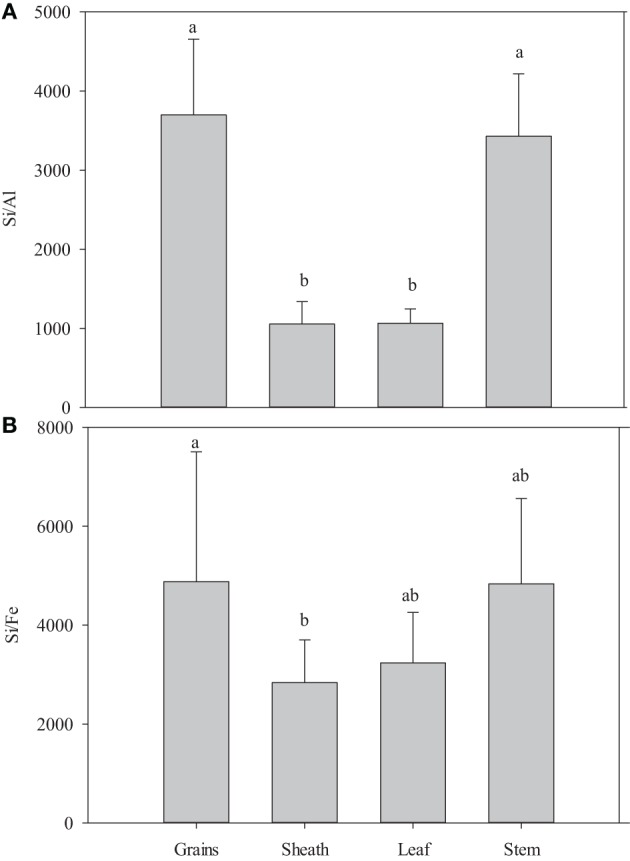
**The ratio of Si/Al **(A)** and Si/Fe **(B)** in phytoliths of five rice cultivars**. Means with various letters are significantly different at the *p* < 0.05 level of confidence according to Duncan's Multiple Range Test.

The mean H_2_O content in phytoliths ranged from 5.4 to 14.5% and showed the following trend: stems > leaves > sheaths = grains. As a result of different hydration rates of phytoliths in the different organs, the SiO_2_ content of phytolith was the highest in the grains (94.71%), then significantly decreased in sheaths (92.50%), leaves (88.47%), and stems (83.04%) (Table 3). The average Al content in phytoliths significantly varied from 0.012 to 0.043%: sheaths and leaves > grains and stems (Table 3; Figure 1A). The average Mg content in phytoliths varied from 0.0043 to 0.0082%: sheaths and leaves = grains > stems (Table 3). The average Fe content in phytoliths ranged from 0.017 to 0.009%: sheaths = leaves = grains = stems (Table 3; Figure 1B). The average OC content in phytoliths varied from 1.93 to 2.46%, without significant change between plant organs (Table 3).

**Table 3:** **Elemental composition (%) of phytoliths extracted from organs of different rice cultivars. Means with various letters are significantly different at the *p < 0.05* level of confidence according to Duncan's Multiple Range Test**.

**Rice cultivars**	**Rice organs**	**SiO_2_**	**Al**	**Mg**	**Fe**	**OC**	**H_2_O^a^**
		**%**					
Xiushui-09	Grains	95.27 ± 1.93A	0.011 ± 0.007A	0.0050 ± 0.0004C	0.005 ± 0.001A	2.11 ± 0.12A	**2.58** ± **1.73AB**
Ning-81		95.08 ± 0.54A	0.011 ± 0.004A	0.0073 ± 0.0007AB	0.012 ± 0.001A	2.89 ± 0.84A	**1.98** ± **0.76B**
Xianghu-301		91.40 ± 1.02A	0.011 ± 0.003A	0.0058 ± 0.0003BC	0.007 ± 0.006A	2.35 ± 0.01A	**6.21** ± **1.44A**
Zhejing-37		95.74 ± 0.29A	0.022 ± 0.004A	0.0051 ± 0.0005C	0.014 ± 0.005A	2.32 ± 0.03A	**1.87** ± **0.41B**
Jiahua-11		96.07 ± 0.89A	0.016 ± 0.005A	0.0081 ± 0.0004A	0.018 ± 0.002A	2.62 ± 0.20A	**1.24** ± **1.26B**
**Mean**		**94.71a**	**0.014b**	**0.0063ab**	**0.011ab**	**2.46a**	**2.78c**
Xiushui-09	Sheath	93.16 ± 0.36A	0.048 ± 0.010A	0.0067 ± 0.0008B	0.014 ± 0.001A	2.03 ± 0.15A	**4.69** ± **0.51AB**
Ning-81		94.31 ± 0.15A	0.041 ± 0.002AB	0.0092 ± 0.0004A	0.012 ± 0.002A	1.96 ± 0.11A	**3.62** ± **0.21B**
Xianghu-301		91.34 ± 0.23A	0.028 ± 0.002B	0.0087 ± 0.0004A	0.013 ± 0.002A	2.05 ± 0.01A	**6.52** ± **0.33A**
Zhejing-37		92.31 ± 1.19A	0.055 ± 0.005A	0.0074 ± 0.0003AB	0.016 ± 0.002A	2.21 ± 0.11A	**5.34** ± **1.68AB**
Jiahua-11		91.40 ± 0.85B	0.043 ± 0.002AB	0.0091 ± 0.0004A	0.030 ± 0.001A	1.42 ± 0.17B	**7.04** ± **1.20A**
**Mean**		**92.50b**	**0.043a**	**0.0082a**	**0.017a**	**1.93a**	**5.44c**
Xiushui-09	Leaf	87.87 ± 2.50AB	0.036 ± 0.010A	0.0057 ± 0.0007B	0.016 ± 0.003AB	1.87 ± 0.31A	**10.16** ± **3.54AB**
Ning-81		93.54 ± 0.98A	0.052 ± 0.003A	0.0105 ± 0.0008A	0.022 ± 0.002A	2.45 ± 0.14A	**3.86** ± **1.39B**
Xianghu-301		83.22 ± 0.84B	0.043 ± 0.002A	0.0092 ± 0.0004A	0.011 ± 0.001A	2.21 ± 0.04A	**14.46** ± **1.19A**
Zhejing-37		87.83 ± 1.87AB	0.034 ± 0.009A	0.0050 ± 0.0004B	0.012 ± 0.003AB	2.57 ± 0.15A	**9.51** ± **2.64AB**
Jiahua-11		89.87 ± 2.43AB	0.034 ± 0.004A	0.0042 ± 0.0004B	0.009 ± 0.004B	2.30 ± 0.04A	**7.75** ± **3.44AB**
**Mean**		**88.47c**	**0.040 a**	**0.0069a**	**0.014ab**	**2.28a**	**9.15b**
Xiushui-09	Stem	80.22 ± 1.63C	0.009 ± 0.005A	0.0053 ± 0.0001A	0.005 ± 0.002B	2.11 ± 0.12C	**17.64** ± **2.31A**
Ning-81		82.14 ± 2.75BC	0.013 ± 0.004A	0.0047 ± 0.0003B	0.010 ± 0.001AB	1.85 ± 0.16C	**15.96** ± **3.89AB**
Xianghu-301		80.75 ± 0.52BC	0.013 ± 0.002A	0.0041 ± 0.0004B	0.010 ± 0.001AB	3.36 ± 0.04A	**15.84** ± **0.74AB**
Zhejing-37		84.74 ± 1.68AB	0.009 ± 0.005A	0.0037 ± 0.0004B	0.007 ± 0.001AB	2.21 ± 0.11C	**13.02** ± **2.38AB**
Jiahua-11		87.37 ± 0.12A	0.015 ± 0.005A	0.0039 ± 0.0004B	0.012 ± 0.002A	2.73 ± 0.15B	**9.85** ± **0.17B**
**Mean**		**83.04c**	**0.012b**	**0.0043b**	**0.009b**	**2.45a**	**14.46a**

*Different lower case and uppercase letters indicate significant differences among the stands in rice organs and rice cultivars*.

a *According to the following equation to estimate the content of H_2_O in phytoliths. H_2_O = 100-SiO_2_-Al_2_O_3_-Fe_2_O_3_-MgO-OC*.

### The release of bio-available Si

**Figure 2:**
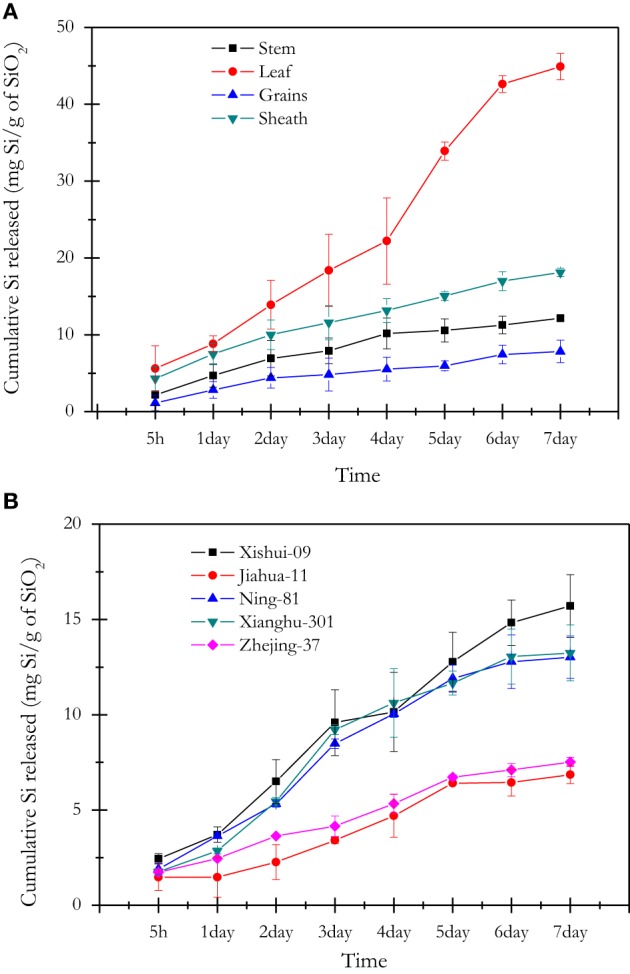
**Cumulative amount of Si released over time per g of SiO_2_ in phytoliths (mg bioavailable Si/g SiO_2_ in phytoliths) by successive extractions with 0.01 M CaCl_2_**. **(A)** Si released from phytoliths extracted from the organs of five rice cultivars (each phytolith sample is the mixture of same organ from five rice cultivars); **(B)** Si released from phytoliths extracted from stems of five rice cultivars. Each point represents the mean of three replicates.

After 7 days, the cumulative release of bio-available Si from phytoliths of different plant organs (mg Si/g SiO_2_ in phytoliths; Figure 2A) significantly varied from 7.8 to 44.9 mg Si/g SiO_2_ and was the highest in leaves (44.9 mg Si/g of SiO_2_), then significantly decreased in sheaths (18.1 mg Si/g of SiO_2_), stems (12.2 mg Si/g of SiO_2_), and grains (7.8 mg Si/g of SiO_2_). After 7 days, the release of bio-available Si from phytoliths of stems of different rice cultivars (mg Si/g SiO_2_ in phytoliths; Figure 2B) significantly varied from 6.9 to 15.7 mg Si/g SiO_2_: Xiushui-09 (15.7 mg Si/g SiO_2_) > Xianghu-301 (13.2 mg Si/g SiO_2_) and Ning-81 (13.0 mg Si/g SiO_2_) > Zhejiang-37 (7.5 mg Si/g SiO_2_) and Jiahua11 (6.9 mg Si/g SiO_2_).

## Discussion

### Impact of rice cultivar and organ on concentration and elemental composition of phytoliths

Our results show that a significant correlation exists between the phytolith content in plants and the elemental concentration in plants for the five rice cultivars (Phytolith vs. Si, *R*^2^ = 0.8895; Phytolith vs. Al, *R*^2^ = 0.6494; Phytolith vs. Fe, *R*^2^ = 0.572) (Table 4). A correlation also exists between the phytolith content in plants and the phytolith elemental content (Phytolith vs. Phytolith-Al, *R*^2^ = 0.6518; Phytolith vs. Phytolith-Fe, *R*^2^ = 0.2973; Phytolith vs. Phytolith-Mg, *R*^2^ = 0.3194) (Table 5). This implies that the uptake of Si Al, Fe may significantly affect phytolith production in plant organs. Previous studies already show that climatic (transpiration flow), pedologic conditions (Si availability) and plant species can influence significantly the uptake of Si and the formation of phytoliths in plants (Bartoli and Wilding, 1980; Epstein, 1999; Piperno et al., 2002; Hodson et al., 2005; Henriet et al., 2008; Cornelis et al., 2010, 2011; Kameník et al., 2013; Li et al., 2013). Our study in identical pedo-climatic conditions shows that the precipitation of elements between plant organs of rice cultivars is also controlled by plant physiology, i.e., more transpiration sites in leaves and sheaths. This explains why the elemental composition in organs is related to elemental composition in phytoliths.

**Table 4:** **Correlation analysis of the composition (elemental concentration and phytolith content) of organs in rice cultivars**.

**Elements**	**Phytolith**	**Si**	**Al**	**Fe**
Phytolith	1	0.8895^**^	0.6494^**^	0.572^**^
Si		1	0.4612^**^	0.4249^**^
Al			1	0.4481^**^
Fe				1

** *p < 0.01; (n = 20)*.

**Table 5:**
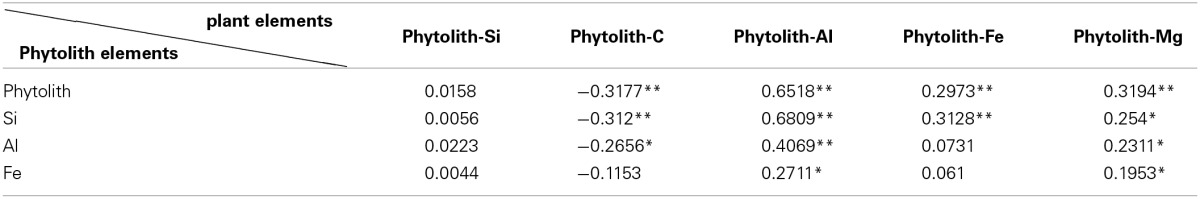
**The correlation between phytolith elements and plant elements**.

^*^p < 0.05; ^**^p < 0.01; (n = 20).

Our study also demonstrates that for rice cultivars, a significant negative correlation exits between OC content of phytoliths and the elemental composition in plants (Phytolith-C vs. Phytolith, *R*^2^ = −0.3177, Phytolith-C vs. Si, *R*^2^ = −0.312, Phytolith-C vs. Al, *R*^2^ = −0.2656) (Table 5). The higher the elemental content in plants is, the higher the phytolith content will be but the amount of occluded OC within the phytoliths will be lower. The different types of phytolith morphology and specific surface area between plant organs (e.g., between rice stem and rice sheath) may imply different occluded-OC content in phytoliths (Li et al., 2013). However, the impact of this factor on the OC occlusion within phytoliths remains to be examined.

### The production and stability of rice phytoliths

**Figure 3:**
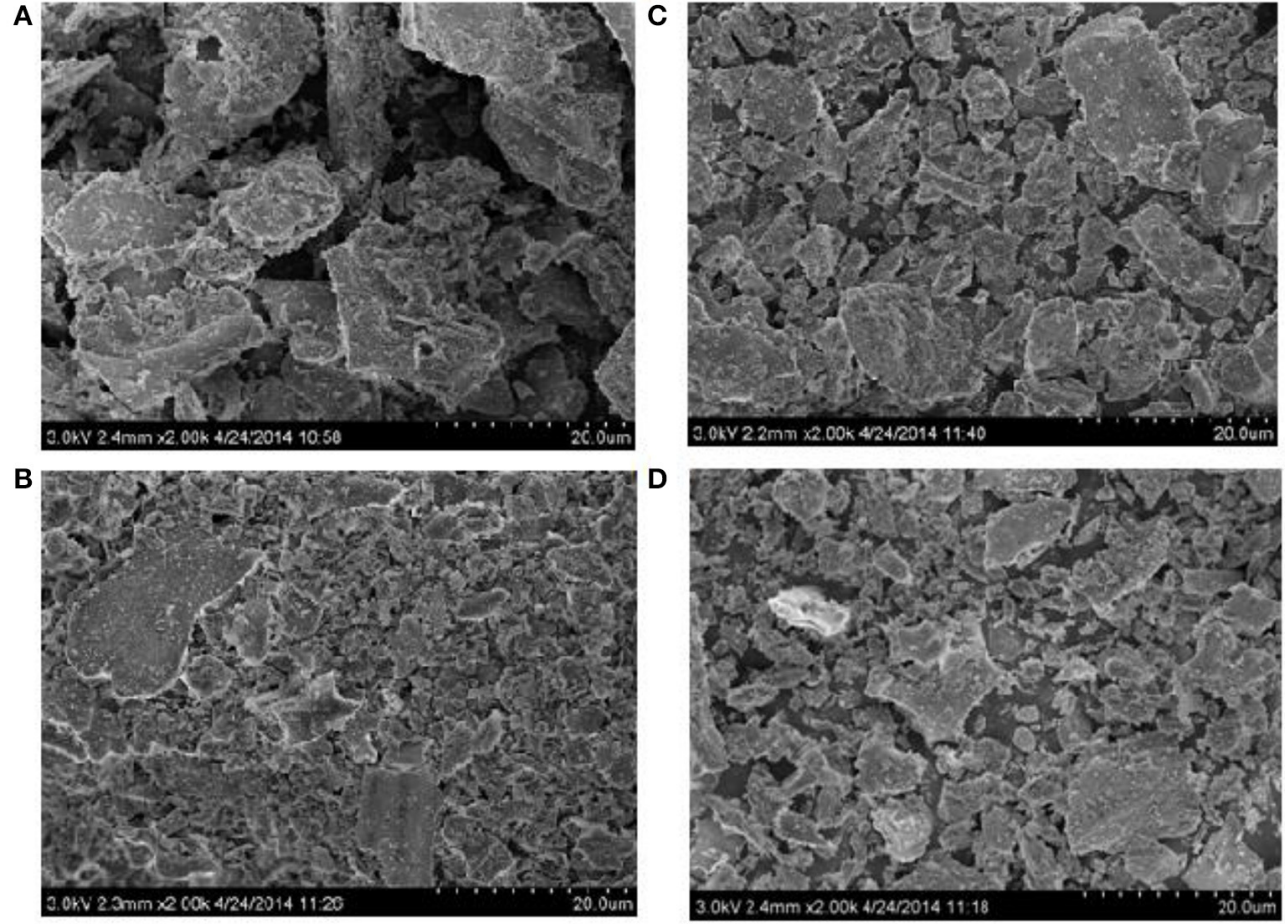
**Representative scanning electron microscope (SEM) images of phytoliths from rice organs (A) grains, (B) leaf, (C) sheath, and (D) stem**.

In the literature, we find some contradictory results about the control of plant species on the dissolution rate of phytoliths. Fraysse et al. (2009) document similar dissolution rates of phytoliths for four different plant species (horsetail, larch, elm and fern), while Bartoli and Wilding (1980) and Bartoli (1985) show that phytoliths from grass plants and deciduous species are more easily dissolved than that of coniferous species because of physico-chemical differences, i.e., higher Si/Al and Si/Fe ratios and water content. The adsorption of Al, Fe and other bi- and trivalent metals on phytoliths may influence their surface properties and decrease their rate of dissolution in the soil environment (Dove, 1995). In this study, the phytoliths of leaves and sheaths in five rice cultivars are characterized by lower ratio of Si/Fe and Si/Al compared to grains and stem (Figures 1A,B), which means that phytolith in leaves and sheaths incorporate more Al and Fe in their structure than in grains and stems. However, we clearly show that the kinetic release of bio-available Si from phytoliths in leaves are much higher relative to phytoliths from sheaths, grains and stems (Figure 1A). This is in contradiction with a previous view showing that phytolith solubility decreases with increasing metal content (Bartoli and Wilding, 1980; Bartoli, 1985; van Bennekom et al., 1989), but not with the results of Fraysse et al. (2009) showing similar dissolution rates for phytoliths with different elemental composition. This can be explained by the fact that phytolith solubility of rice cultivars could be controlled more by differences in terms of morphology (i.e., specific surface area) (Figure 3) and hydration rate (Table 3) than chemical composition. Our data show that the hydration rates of phytoliths (highest H_2_O content in stem phytoliths) are not related to Si bio-availability which indicates that hydration rate can only explain a small part of the variation in phytolith solubility. This observation requires further investigations for better quantifying the impact of morphology and hydration rate on dissolution rates of phytoliths from different rice cultivars. The Figure 1B also shows that the release of bio-available Si from phytoliths in stems among different rice cultivars vary significantly. Thus, besides the impact of rice organs on phytolith solubility, we show that the genetics also partly governs the release of bio-available Si from phytoliths.

Phytoliths are a readily soluble, potential Si source for plants (Berthelsen et al., 2001; Sauer et al., 2006; Cornelis et al., 2011). Recent research also indicate that organic matter pyrolyzed from plants (biochar) with high phytolith content can be applied as a potential source of bio-available Si for crops of Si-accumulator plants (Houben et al., 2014; Liu et al., 2014). Therefore, amendment with biochar made from leaves and sheaths of rice characterized by the highest amount of phytoliths and the highest release rate of Si could be promising for enhancing the bio-availability of Si while increasing OC storage and soil fertility.

The findings of this study suggest that the optimization of utilization of different plant organs as Si amendment could play a key role for more efficient productivity in high Si-accumulating plants. On the other hand, we should increase PhytOC content in soils by increasing return of plant organs characterized by lower phytolith solubility such as grains and stems. We thus suggest further study of the kinetic release of dissolved Si and dissolved OC and the evolution of Si and OC stock in soils after amendment with different plant organs.

## Conclusions

The present study mainly focused on the elemental compositions of organs and phytoliths in rice of different cultivars. The rice cultivar significantly influences the elemental composition of plant organs and phytoliths. Our results show that rice cultivar and Si, Al, Fe uptake may impact production of phytoliths in plants and their quality (chemical composition). The OC content of phytoliths in rice cultivars seems to be significantly affected by the content of Si, Al, and Fe in plants, but not by the elemental concentrations in phytoliths.

In different rice cultivars, the role of the morphology and hydration rate of phytoliths seems to be at least as important as the chemical composition. This documents that rice organs and cultivar can largely influence Si dynamics in soil-plant systems through variable solubility of phytoliths. The soil amendment with plant residues and/or plant biochar should therefore be carried out taking into account the phytolith solubility of different plant organs of Si-high accumulator plants (e.g., leaves and sheaths of rice). On the other hand, phytoliths from grains and stems of rice seem to be more stable in soil and should be used if we aim to increase the soil OC stock through higher PhytOC. A more efficient use of plant residue (type of plant organ) as soil amendment should be considered to improve agronomical productivity of Si-high accumulating plants.

### Conflict of interest statement

The authors declare that the research was conducted in the absence of any commercial or financial relationships that could be construed as a potential conflict of interest.

## Footnotes

^1^Available online at: http://ricestat.irri.org:8080/wrs2/entrypoint.htm.

^2^Available online at: http://en.wikipedia.org/wiki/FAO_soil_classification
